# Intra-Accumbens Baclofen, But Not Muscimol, Increases Second Order Instrumental Responding for Food Reward in Rats

**DOI:** 10.1371/journal.pone.0040057

**Published:** 2012-07-09

**Authors:** Kim G. T. Pulman, Elizabeth M. Somerville, Peter G. Clifton

**Affiliations:** 1 School of Psychology, University of Sussex, Brighton, Sussex, United Kingdom; 2 School of Life Sciences, University of Sussex, Brighton, Sussex, United Kingdom; University of Chicago, United States of America

## Abstract

Stimulation of either GABA_A_ or GABA_B_ receptors within the nucleus accumbens shell strongly enhances food intake in rats. However the effects of subtype-selective stimulation of GABA receptors on instrumental responses for food reward are less well characterized. Here we contrast the effects of the GABA_A_ receptor agonist muscimol and GABA_B_ receptor agonist baclofen on instrumental responding for food using a second order reinforcement schedule. Bilateral intra-accumbens administration of baclofen (220–440 pmol) stimulated responding but a higher dose (660 pmol) induced stereotyped oral behaviour that interfered with responding. Baclofen (220–660 pmol) also stimulated intake of freely available chow. Muscimol (220–660 pmol) was without effect on responding for food on this schedule but did stimulate intake of freely available chow. Unilateral administration of either baclofen or muscimol (220 pmol) induced similar patterns of c-*fos* immunoreactivity in several hypothalamic sites but differed in its induction in the central nucleus of the amygdala. We conclude that stimulation of GABA_A_ or GABA_B_ receptors in the nucleus accumbens shell of rats produces clearly distinguishable effects on operant responding for food.

## Introduction

The nucleus accumbens is a key structure within neural systems that have an established role in feeding. Pharmacological evidence suggests specific and dissociable roles for dopamine, opioid peptides, acetylcholine and amino acids in controlling sub-components of food motivated behaviours [1]. GABA in the accumbens shell plays a fundamental role in the modulation of food intake; inhibition of GABA metabolism, leading to increased local concentrations of GABA, stimulates feeding behaviour [2]. Intra-accumbens administration of GABA agonists also leads to a significant selective increase in short-term intake [2]–[5].

The accumbens receives numerous inputs signalling the presence and location of reward-related stimuli and is thought to mediate response selection and/or adaptive switching [6]–[8]. Electrophysiological studies demonstrate that response to reward-related stimuli is associated with inhibition of a subset of accumbens neurons during appetitive and consummatory behaviors [9], [10]. These authors suggest that the sustained inhibition of GABA-ergic projection neurons, perhaps through activation of fast-spiking GABA-ergic interneurons [11], disinhibits target regions, permissively gating and maintaining feeding behaviors. Disinhibition of a lateral hypothalamic target is believed to be crucial to GABA stimulation of feeding through a direct or indirect pathway [4].

Evidence for increased feeding following pharmacological activation of accumbens GABA_A_ receptors is almost entirely restricted to consummatory motor actions [12], [13]. Kelley and colleagues [1] hypothesise that this arises through activation of a subset of hypothalamic behavioral control columns [14] directly exciting relevant hindbrain motor pattern generators. Wirtshafter and Stratford [15], [16] have recently demonstrated that intra-accumbens administration of the GABA_A_ agonist muscimol can also enhance responding for food on both a progressive ratio schedule and on a fixed ratio schedule.

There is little evidence to suggest that GABA_A_ and GABA_B_ agonists infused into the accumbens shell exert differential effects on either the appetitive or consummatory components of feeding behavior [2], [5] or that they have other differential behavioural effects. However we have shown that the effects of intra-accumbens infusions of either the GABA_A_ receptor agonist muscimol or the GABA_B_ receptor agonist baclofen on feeding-related behaviours can be distinguished [17]. The data suggested that the action of baclofen mimics more ‘natural’ motivational manipulations such as a short period of food withdrawal.

The studies reported here investigated the effects of bilateral intra-accumbens administration of muscimol or baclofen on instrumental responding for food using a second order schedule which includes an initial period of responding without reward followed by a longer phase in which food is available [18]. Subjects in the second and third experiments received a final unilateral administration of either baclofen or muscimol. Selected brains were subsequently processed to visualise c-*fos* immunoreactivity as a measure of neuronal activity in different brain areas relevant to the induction of feeding behaviour [4], [19].

## Results

### Experiment 1

**Figure 1:**
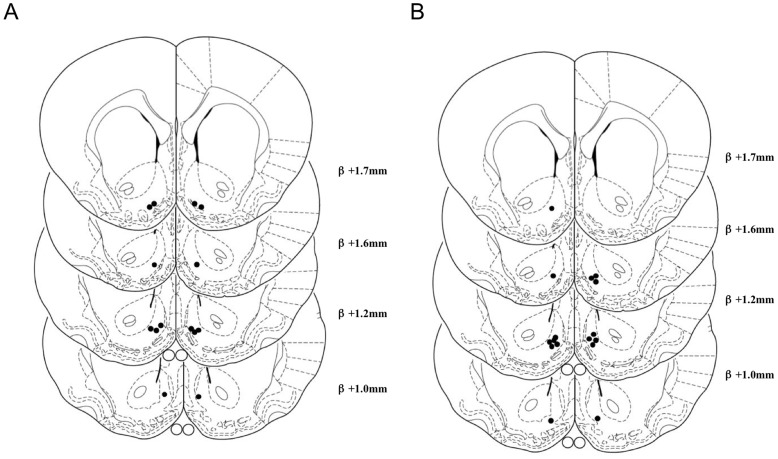
Infusion sites for baclofen. Infusion sites for animals used in Experiment 1 (A) and Experiment 2 (B), plotted on drawings taken from Paxinos and Watson [58].

Seven animals had bilateral placements within the shell of the nucleus accumbens (Figure 1A) and the remaining five did not meet the criteria for bilateral placements and were excluded from further analysis. The free intake test (440 pmol baclofen) revealed a significant stimulation of chow consumption by GABA_B_ receptor stimulation (saline: 2.1 g±0.27; baclofen: 4.6 g±0.38, *P*<0.001).

**Figure 2:**
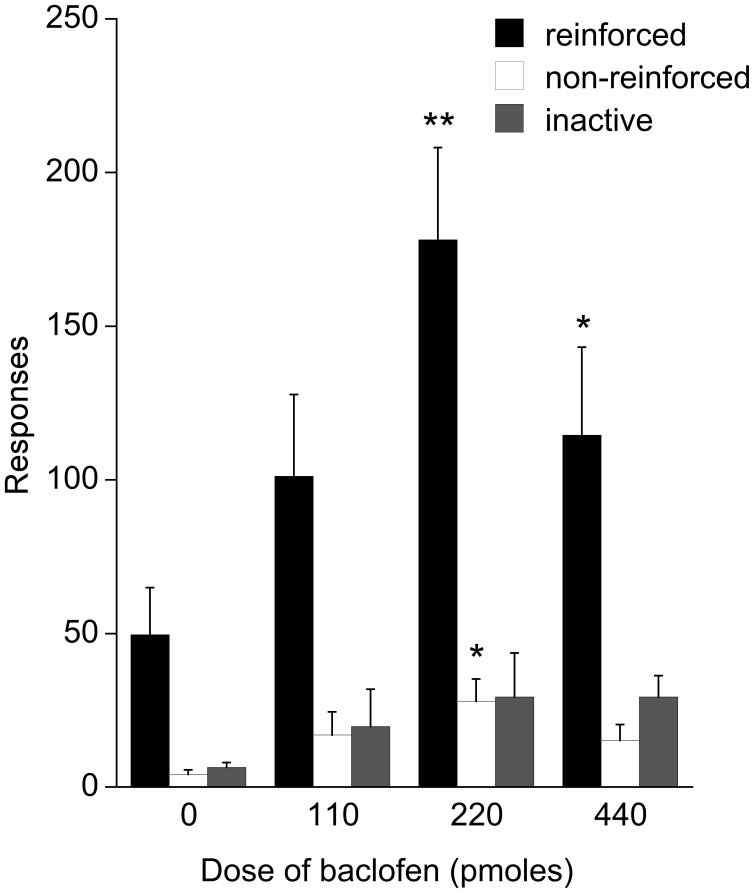
Operant responding following baclofen infustion. Reinforced and non-reinforced active lever, and inactive lever responding in a 30 minute test session following bilateral intra-accumbens infusion of baclofen in Experiment 1.

Baclofen increased reinforced responses on the second order schedule (Fig. 2: *F*_3,18_ = 10.95, *P*<0.001). The effect was maximal at 220 pmol and significantly greater at this dose than at either 110 or 440 pmol (p<0.05). Non-reinforced responses were also increased at 220 pmol baclofen (Fig. 2: *F*_3,18_ = 4.03, *P*<0.05) There was no effect on responding on the inactive lever (Fig. 2: *F*_3,18_ = 1.62, NS).

### Experiment 2

Eight animals had bilateral placements within the shell of the nucleus accumbens (Figure 1B) and the remaining four did not meet the criteria for bilateral placements and were excluded from further analysis. 660 pmol baclofen resulted in a significant stimulation of free chow intake (saline: 1.9 g±0.17; baclofen: 4.8±0.54, *P*<0.001).

**Figure 3:**
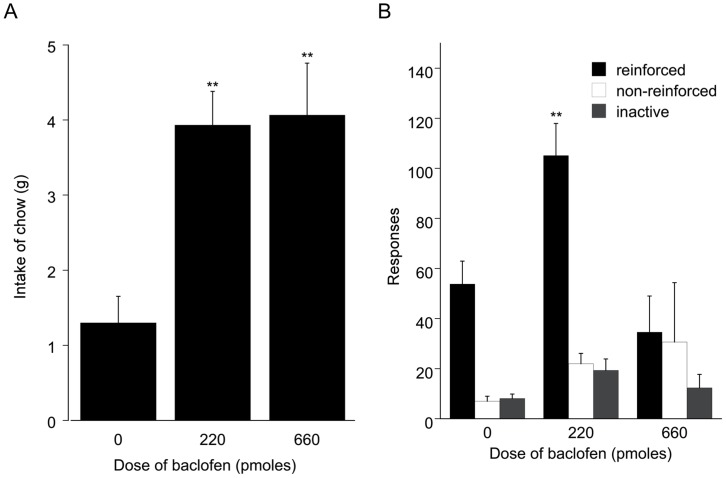
Food intake and operant responding following baclofen infusion. A: Intake of freely available chow in a 30 minute test session following bilateral intra-accumbens infusion of baclofen in Experiment 2. B: Reinforced and non-reinforced active lever, and inactive lever responding in a 30 minute test session following bilateral intra-accumbens infusion of baclofen in Experiment 2.

**Figure 4:**
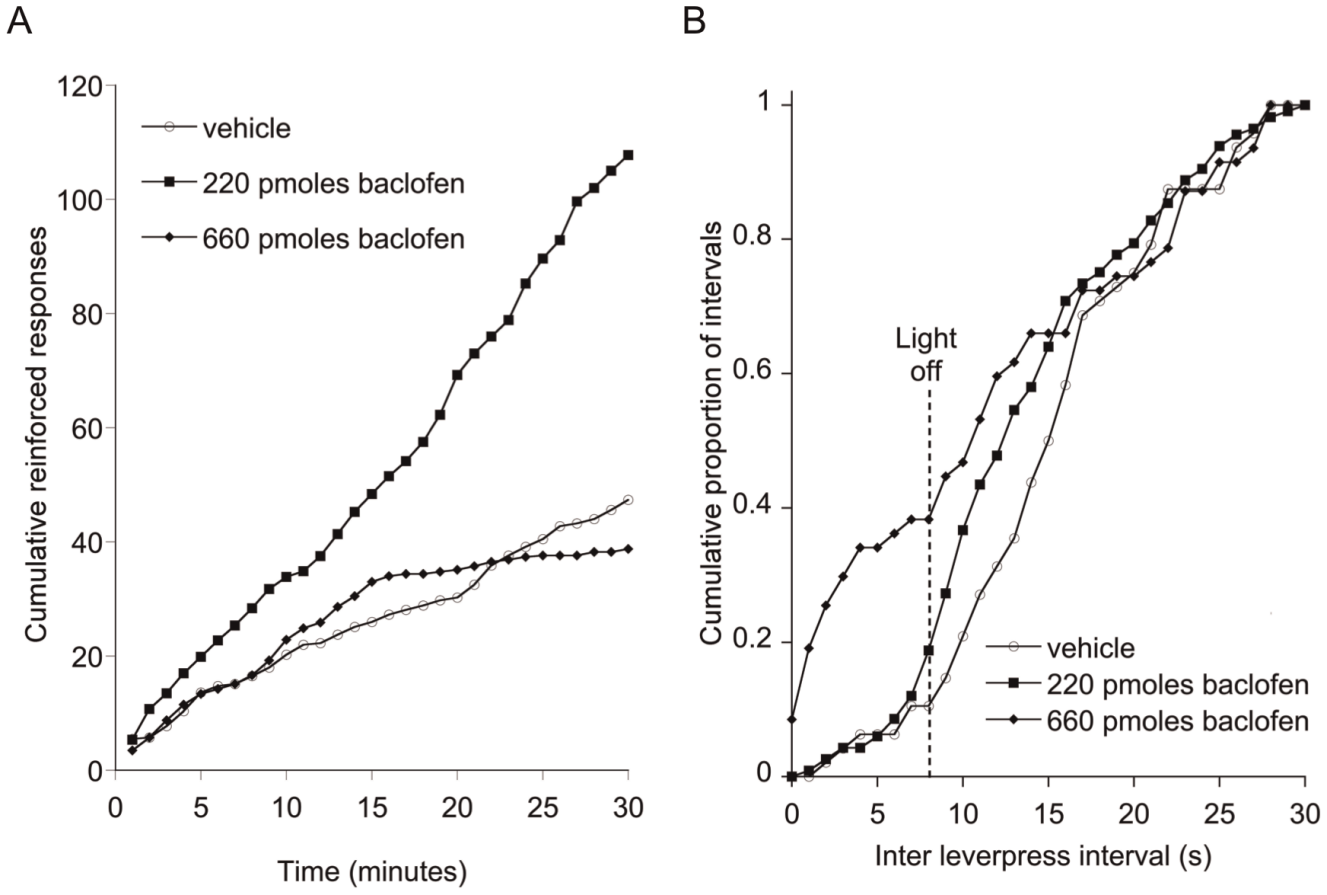
Temporal structure of operant responding following baclofen infusion. Effects of bilateral intra-accumbens infusion of baclofen (Experiment 2) on A. Cumulative lever presses, B. inter – lever press intervals (5^th^ to 1^st^ press on FR5 requirement).

Baclofen increased reinforced responses on the second order schedule (Fig. 3B: *F*_2,16_ = 14.44, *P*<0.001), but only at the 220 pmol dose. There was no effect of baclofen on either non-reinforced responses or responses on the inactive lever in this experiment. A plot of cumulative responses (Fig. 4A) demonstrated a uniform enhancement of responding throughout the test session and did not suggest a differential enhancement of responding in either the initial appetitive or the later consummatory component of the schedule. This was confirmed by an analysis of response rates in the appetitive (first 5 minutes) and consummatory (remaining 25 minutes) phases of the test session (Table 1). The response rate following infusion of 220 pmol baclofen was higher in both the appetitive (*P*<0.05) and consummatory (*P*<0.01) phase than in animals infused with vehicle. Figure 4B plots the distribution of intervals between the 5th and 1st lever presses of the schedule. It demonstrates that the animals normally paused in their responding as the light illuminated after the fifth lever press. The next response was typically delayed until after the light was extinguished. Intra-accumbens infusion of 220 pmol baclofen was associated with a small leftward shift of the distribution, indicating a more rapid return to lever pressing but only after the light was extinguished. Infusion of 660 pmol baclofen, by contrast, resulted in 3/8 animals emitting 10 or fewer lever presses in the entire session. Responding by the remainder was also at a low level (Figure 3B) and such responses frequently occurred while the light was still illuminated (Figure 4B).

**Table 1:** Rates of responding in the appetitive and consummatory phases of Experiments 2 and 3.

Treatment	Phase	Rate (responses/minute)
Vehicle	Appetitive	2.73±0.49
	Consummatory	1.35±0.25
220 pmol baclofen	Appetitive	4.60±0.35*
	Consummatory	3.74±0.51**
660 pmol baclofen	Appetitive	2.68±0.68
	Consummatory	1.02±0.54
Vehicle	Appetitive	1.55±0.41
	Consummatory	1.21±0.41
220 pmol muscimol	Appetitive	2.28±0.33
	Consummatory	1.03±0.29
440 pmol muscimol	Appetitive	1.48±0.40
	Consummatory	1.41±0.49
660 pmol muscimol	Appetitive	1.28±0.22
	Consummatory	1.08±0.32

Significant differences:

* p<0.05 compared with vehicle.

** p<0.01 compared with vehicle.

The detailed video analysis of the instrumental test sessions revealed that infusion of 660 pmol baclofen, in addition to reducing instrumental responses, also led to a marked increase in oral stereotypy and a substantial decrease in both active behaviour (typically locomotor responses) and rearing (Table 2: dose x behaviour interaction F_8,56_ = 6.27, *P*<0.001). The oral stereotypy was primarily manifested as licking and chewing of the mesh flooring of the cage, but was also sometimes directed towards the response levers or food magazine. The stereotypy also altered the form of grooming responses, with a breakdown of the usual syntactic chain [20]. Instead 6/8 animals predominantly groomed either the tail or genitals. None of these patterns of changed response were apparent at a dose of 220 pmol baclofen; the only change at this dose was an increase in time spent at the magazine as a consequence of increased feeding (data not shown), which is consistent with the data for instrumental responses. There were no obvious rostrocaudal differences in evoked behaviour, and aversive responses (e.g. defensive treading) were not apparent in this test situation.

**Table 2:** Effects of bilateral intra-accumbens infusion of saline, baclofen (660 pmol) or muscimol (660 pmol) on the duration (s) of different behaviour patterns observed during operant test sessions.

vehicle	baclofen	BEHAVIOUR	vehicle	muscimol
16.6±3.9	300±103**	***Oral***	4.7±2.6	20.8±5.1*
1142±46.1	565±106**	***Active***	1126±75.4	1059±85.6
182±32.3	107±45.1	***Rear***	156±33.8	208±37.2
83.6±13.3	73.7±31.5	***Lever***	91.3±16.9	68.8±15.2
89.9±20.5	74.3±27.7	***Magazine***	68.2±27.9	56.8±11.8
40.9±12.2	27.4±22.3	***Ingest***	29.2±19.6	31.5±16.3
155±33.2	299±128	***Groom***	182±24.1	154±20.9
83.3±39.9	330±147	***Inactive***	148±64.7	213.5±9.7
24.6±16.1	79.4±38.3	***Off***	4.8±4.8	2.6±1.3

*Behavior categories.* Oral: oral stereotypies, including repeated licking and mouthing, but not of the body; Active: rapid moving around the test chamber; Rear: body supported on rear limbs only, but forelimbs may be against the sides of the test chamber; Lever: pressing either operant lever; Magazine: head inserted into the food magazine; Ingest: holding and eating a food pellet; Groom: body care movements using the mouth or forelimbs; Inactive: body supported on all four limbs with no locomotor movement; Off: not visible on screen.

* p<0.05 compared with vehicle.

** p<0.01 compared with vehicle.

In the subsequent free intake tests, baclofen stimulated chow consumption at both 220 and 660 pmol (Figure 3A: F_2,16_ = 14.53, *P*<0.001). The stimulation of intake at 660 pmol was similar (4.1 g±0.69) to that observed in the probe test that preceded the tests of instrumental responding.

Unilateral infusion of 0.5 µl of 220 pmol/µl baclofen was associated with a marked increase in c-*fos* immunoreactivity in most, though not all, of the brain areas that were sampled (Table 3: side x area interaction F_9,36_ = 27.9, *P*<0.001). The cell count was elevated ipsilaterally in all sampled areas with the exception of the basolateral nucleus of the amygdala (Table 3).

**Table 3:** Effects of unilateral intra-accumbens infusion of baclofen (220 pmol) or of muscimol (220 pmol) on c-*fos* activity in selected brain regions.

vehicle	baclofen	BRAIN AREA	vehicle	muscimol
34.2±3.4	51.8±4.6*	***VP***	31.6±6.8	49.2±8.2**
170.0±33.0	449.8±46.6**	***LH1***	135.2±20.6	521.4±66.5**
122.8±10.5	299.8±21.8**	***LH2***	122.0±10.5	269.4±13.4**
97.6±10.2	227.0±15.3**	***LH3***	101.0±10.4	227.0±29.8**
114.0±7.6	229.0±15.0**	***LH4***	118.4±11.3	183.6±17.4*
109.0±5.9	157.6±9.1**	***Arc***	157.2±11.5	204.2±16.1
211.8±25.5	310.0±49.0*	***PVN***	222.2±14.7	347.4±35.4**
99±24.8	137.0±23.1*	***CeA***	82.2±7.2	81.8±4.4
71.4±5.9	106.6±12.6	***BLA***	71.8±11.5	74.6±9.5
77.0±10.3	123.2±19.4*	***VTA***	43.8±12.0	84.6±11.7*

Counts of labelled neurons in each of the brain regions, given for (left columns) the vehicle infused side and the baclofen infused side and (right columns) the vehicle infused side and the muscimol infused side. Significant differences from vehicle are shown:

* p<0.05;

** p<.01.

The areas investigated were VP (ventral pallidum at β–0.26 mm); LH1 (lateral hypothalamus at β–1.6 mm); LH2 (lateral hypothalamus at β–2.6 mm); LH3 (lateral hypothalamus at β–3.6 mm); LH4 (lateral hypothalamus at β–4.3 mm); Arc (arcuate nucleus at β–2.8 mm); PVN (paraventricular nucleus of the hypothalamus at β–1.88 mm); CeA (central nucleus of the amygdala at β–3.14 mm); BLA (basolateral nucleus of the amygdala at β–3.3 mm); VTA (ventral tegmental area at β–5.3 mm). All coordinates from Paxinos and Watson [58].

### Experiment 3

**Figure 5:**
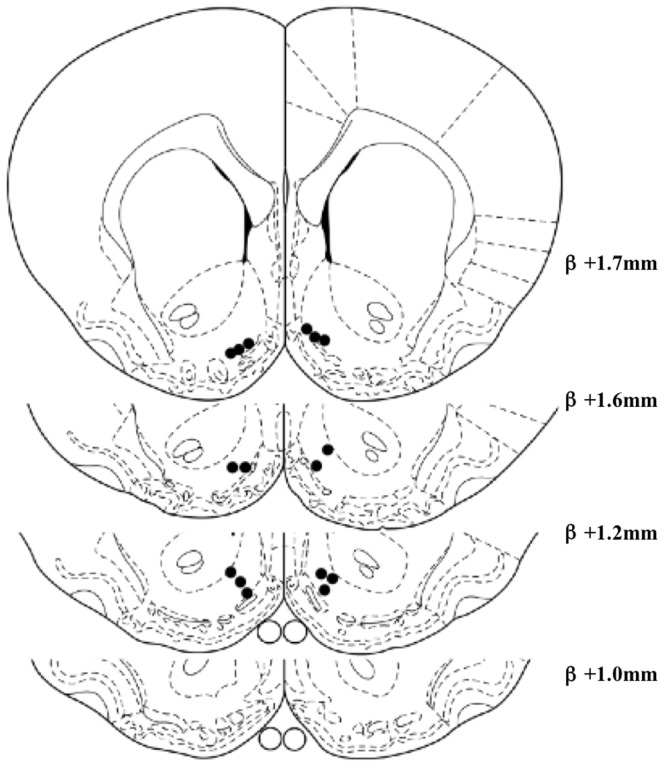
Infusion sites for muscimol. Infusion sites for animals used in Experiment 3, plotted on drawings taken from Paxinos and Watson [58].

Eight animals had bilateral placements within the shell of the nucleus accumbens (Figure 5) and the remaining four did not meet the criteria for bilateral placements and were excluded from further analysis. The initial infusion of 660 pmol muscimol stimulated chow intake (saline: 1.98 g±0.27; muscimol: 5.61 g±0.58; *P*<0.001).

**Figure 6:**
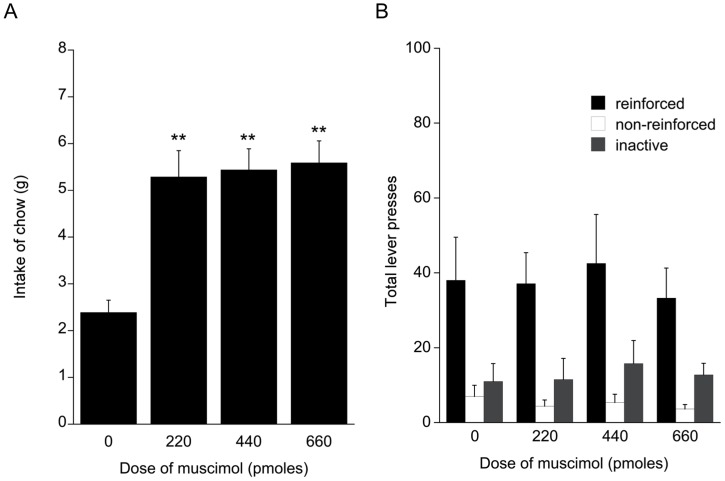
Food intake and operant responding following baclofen infusion. A. Intake of freely available chow in a 30 minute test session following bilateral intra-accumbens infusion of muscimol. B. Reinforced and non-reinforced active lever, and inactive lever responding in a 30 minute test session following bilateral intra-accumbens infusion of muscimol.

Intra-accumbens infusion of muscimol had no effect on responding on the second order schedule (Figure 6B; F_3,21_<1) for reinforced, non reinforced responses or responses to the inactive lever. There was no evidence of responding being suppressed below baseline at higher doses of muscimol and the specificity of responding to the active lever was also maintained. There was also no evidence for any differential effect of muscimol on responding during the appetitive and consummatory phases of the test sessions (Table 1).

Statistical analysis of the detailed video data from the instrumental test sessions using vehicle and 660 pmol muscimol revealed no significant interaction between dose and class of behaviour (Table 2: F_8,56_ = 0.33, NS), although there was a marginally significant increase in oral sterotypy at a dose of 660 pmol muscimol (Table 2). As in Experiment 2, there were no obvious rostrocaudal differences in evoked behaviour, and aversive responses (e.g. defensive treading) were absent.

Intra-accumbens infusion of muscimol resulted in a dose related increase in food intake over 30 minutes (Figure 6A: F_3,21_ = 13.76, *P*<0.001). The stimulation of chow intake at 660 pmol muscimol was 5.6 g±0.47, similar to that recorded in the probe test preceding the tests on the instrumental schedule.

**Figure 7:**
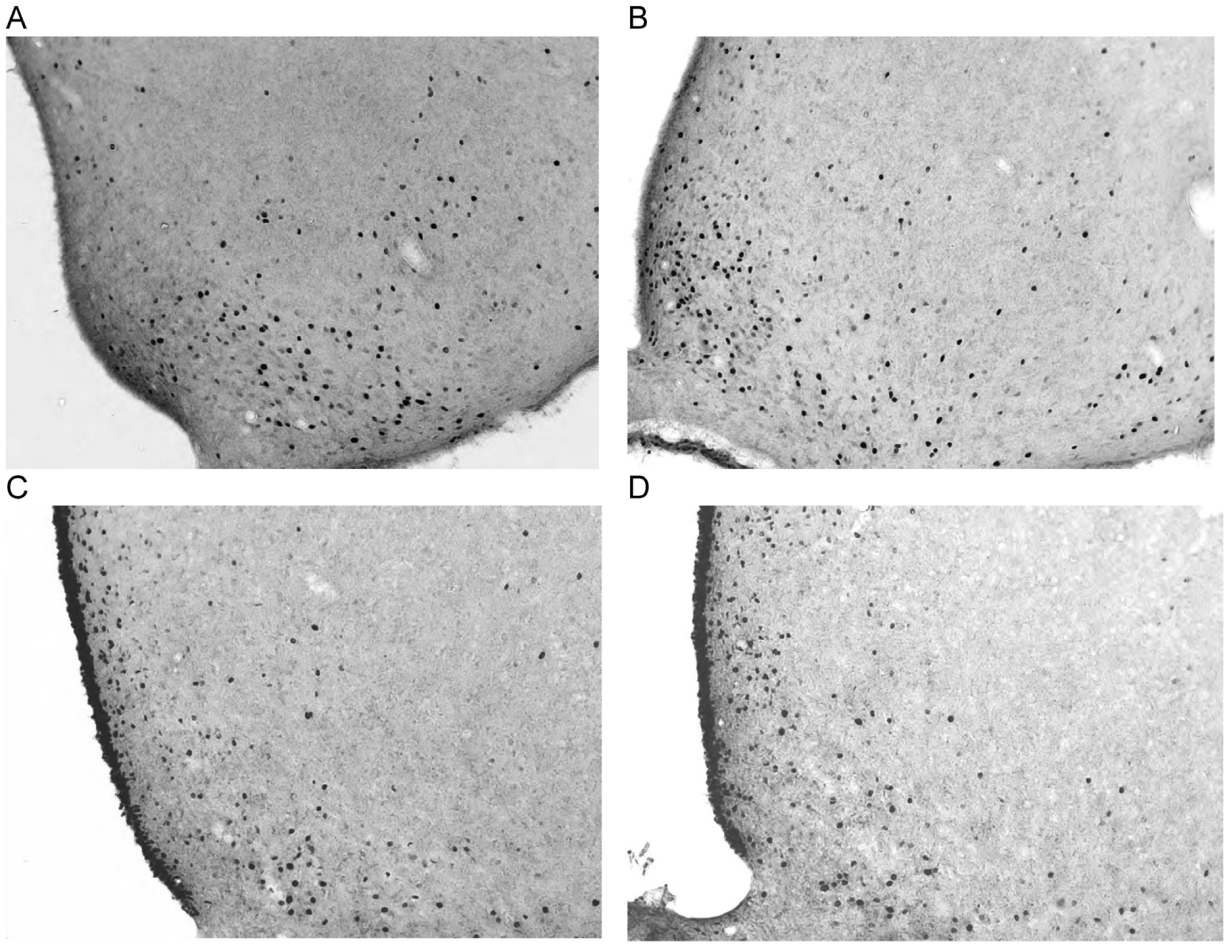
C-*fos* response in arcuate nucleus. Representative sections illustrating c-*fos* immunoreactivity in the arcuate nucleus of the hypothalamus following intra-accumbens infusion with A: saline (baclofen control); B: 220 pmol baclofen; C: saline (muscimol control); D: 220 pmol muscimol. All sections are x100 magnification and β–2.8.

**Figure 8:**
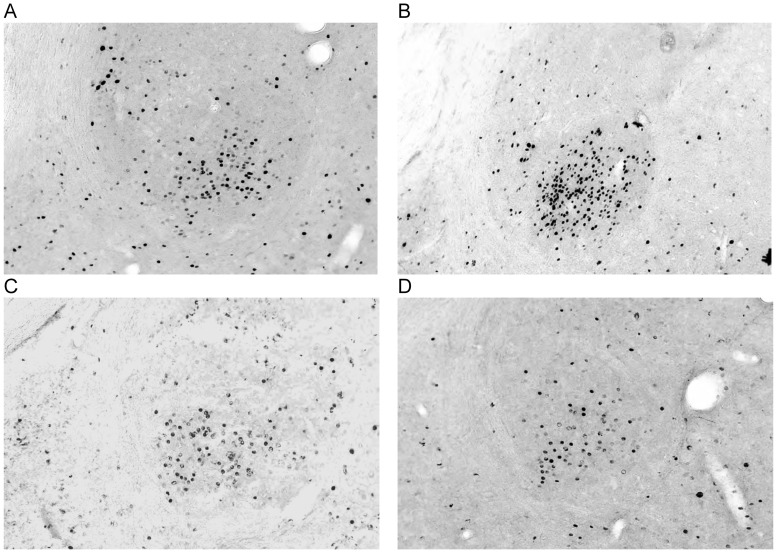
C-*fos* response in central nucleus of the amygdala. Representative sections illustrating c-*fos* immunoreactivity in the central nucleus of the amygdala following intra-accumbens infusion with A: saline (baclofen control); B: 220 pmol baclofen; C: saline (muscimol control); D: 220 pmol muscimol. All sections are x100 magnification and β –3.14.

Unilateral infusion of 0.5 µl of 220 pmol/µl muscimol was associated with a marked increase in c-*fos* immunoreactivity in most brain areas (Table 3: side x area interaction F_9,36_ = 24.4, *P*<0.001). The cell count was elevated ipsilaterally in all brain areas sampled with the exception of the the arcuate nucleus of the hypothalamus (Table 3, Figure 7) and the basolateral and central nuclei of the amygdala (Table 3, Figure 8).

Although Experiments 2 and 3 were not designed to allow explicit contrast of c-*fos* immunoreactivity as a consequence of intra-accumbens infusion of either baclofen or muscimol, a preliminary analysis was conducted. The counts on the vehicle-infused sides were very similar for the two drugs, with no main effect or interaction in an ANOVA restricted to these data (Table 3: F’s <1). There was a significant experiment x drug x side interaction for the full data set (F_9,72_ = 2.58, *P* = 0.012). When restricted by brain area there was a single significant interaction for the central nucleus of the amygdala (F_1,8_ = 8.13, *P* = 0.021) that resulted from increased ipsilateral activation by baclofen, but not by muscimol (Table 3).

## Discussion

Experiments 1 and 2 demonstrated that intra-accumbens administration of the GABA_B_ receptor agonist baclofen (220–440 pmol) enhances instrumental responding for food on a second order reinforcement schedule and that a higher dose of baclofen (660 pmol) induced premature non-reinforced responding and also led to high levels of oral stereotypy. Baclofen stimulated the intake of freely available chow in both experiments. Experiment 3 demonstrated that equivalent doses of the GABA_A_ receptor agonist muscimol did not facilitate responding on the second order schedule although chow intake was increased.

Our results raise several methodological questions. First, the absence of any obvious rostrocaudal pattern of responding and of aversive responses may seem unexpected [21]. However the majority of the placements for infusions were in the rostral part of the accumbens, anterior to the location where aversive reactions to muscimol infusion have been observed [21]–[23]. In addition our animals had very extensive habituation to the test environments, both prior to and following surgery, and we were also using a different strain of rat than these earlier studies. Second, we used a relatively large number of infusions (typically 8–10) in each animal. However our experimental design for experiments 2 and 3 incorporated an intake test with the same drug dose at the very beginning and end of testing for each animal. The data demonstrated that there was no change in responsiveness over this period.

Several possible explanations for the enhancement of instrumental responding by baclofen can be discounted. Psychomotor activation is unlikely since we observed no change in active behaviour at 220 pmol baclofen and suppression at the higher dose of 660 pmol. Failure of inhibitory control is also unlikely since 220 pmol baclofen was not associated with any loss of selectivity between responding to the active and inactive levers and did not lead to premature responding on the active lever while the CS was still illuminated (Figure 4B). Earlier studies have implicated the accumbens core, rather than the shell, in the modulation of impulsive control and response to delayed reinforcement [24]. Although a simple motivational enhancement would be consistent with the data for baclofen, taken in isolation, it would not explain why there is a dissociation between the effects of baclofen and muscimol on instrumental responding despite similar increases in consumption during the free intake tests.

The stimulatory effect of 220 pmol baclofen on instrumental responding might arise from an enhancement of the hedonic properties of either the primary reinforcer (food) or of the visual CS. The former seems unlikely since instrumental responding increased even during the appetitive phase of the schedule (Figure 4A, Table 1). There is evidence that a CS associated with a palatable reinforcer can evoke positive hedonic responses [25], but such effects are only evident after a small number of pairings of CS and reinforcer and are greatly diminished or absent following more extended training [26].

Finally, the stimulatory effect of 220 pmol baclofen on instrumental responding might result from an increase in the salience, or incentive value, of the cues provided by the schedule, accounting for the more rapid return to lever pressing at the offset of the light CS (Figure 4B) and a uniform increase in lever pressing during both phases of the schedule (Figure 4A). It is also consistent with unchanged responding on the inactive lever or in non-reinforced lever presses while the visual CS is illuminated (Figure 2, 3B). This explanation is attractive because of the similarities between the behavioural effects of electrical stimulation of lateral hypothalamus and GABA-ergic stimulation of the accumbens [2] and the arguments in favour of an incentive-based explanation in the former case [27]. It is possible that this effect might also oppose any development of satiety later in the test session. The response rate of animals infused with vehicle decreases after 15 minutes which might indicate the development of satiety. However the actual amount of of food ingested by this point is relatively small, and is unlikely to exceed 0.7 g, which is less than half the amount of increase in free intake at this dose in an earlier study [17].

In Experiment 3 muscimol was without effect on instrumental responding in the second order schedule. Zhang et al [12] reported that muscimol (350–1750 pmol) did not enhance responding on an arithmetic progressive ratio (PR2) schedule although there was some evidence of increased responding at 876 pmol. Wirtshafter and Stratford [15], [16] have recently shown that this dose significantly enhances responding on both an arithmetic PR6 and a FR5 schedule. Our pilot observations indicated that doses at these higher levels induced significant motor side effects that interfered with responding, probably because the Lister hooded strain of rat used here is more sensitive to such effects than the Sprague Dawley strain used in earlier studies. One critical difference between progressive ratio and second order schedules, especially the one used in the present study, is that reinforcement density is high at the beginning of the progressive ratio test session and then declines, whereas the opposite is the case with the schedule used here. Reinforcement density is consistently high in a fixed ratio schedules. These differences suggest that baclofen enhances responding, at least in part, through increasing the incentive value of the CS associated with lever pressing whereas muscimol has a broader motivational effect that is more dependent on immediate reinforcement from the food reward.

The detailed behavioural observations made in Experiments 2 and 3 revealed evidence of oral stereotypy and abnormal grooming patterns following 660 pmol baclofen (Table 2) sufficient to interfere with instrumental responding, thus leading to an inverted ‘U’ dose response function (Figures 2 and 3B). Aspects of this behaviour, especially the repetitive grooming of the tail and also of the genital area, resemble that described following electrical stimulation of the lateral hypothalamus [28] and was absent following infusion of muscimol (Table 2).

Baclofen and muscimol produced similar c-*fos* activation patterns (Table 3), with the exception of the arcuate nucleus of the hypothalamus and the central nucleus of the amygdala (Figures 7 and 8), broadly comparable to those reported for muscimol infused unilaterally [19] and bilaterally [4]. The output of the accumbens shell is from the GABAergic medium spiny neurons [29] which project ipsilaterally [30], [31]. Infusion of GABA agonists will release target areas from inhibition, thus giving rise to elevated levels of c-*fos* immunoreactivity. Unilateral infusions also distinguish between neuronal activation resulting from the drug infusion from any non-specific activation due to the infusion procedure and associated handling [19].

The increase in LH c-*fos* supports the model advanced by Kelley et al [1], whereby inactivation of the accumbens shell leads to activation of the LH which, when transmitted to the relevant behavioural control column activates approach to food and feeding [14]. We observed this pattern of c-*fos* activation after infusion by either drug, at a dose which produced the feeding response without any gross alterations in motor behavior.

The rostrocaudal pattern of activation reported here within LH is very similar to that reported by Stratford [19]. Some of the LH activation is in sub-regions from which feeding may be elicited. Duva et al [32] have shown in the rat that there are afferents from the nucleus accumbens to the tuberal region of LH where NMDA elicits eating. Baldo et al [33] have shown that muscimol infused into the nucleus accumbens shell results in the activation of c-*fos* within orexin/hypocretin cells in the perifornical area of the LH. Within the LH, it seems quite probable that much of the c-*fos* activation is trans-synaptic. Glutamate is most probably the transmitter which gives rise to the excitation of the LH neurons, and hence c-*fos* activation, as it has been shown by Stanley et al [34], [35] to be the key neurotransmitter within the hypothalamus in terms of the activation of feeding behavior.

Both the PVN and the arcuate are considered to be involved in the regulation of feeding [36]. Both these nuclei receive inputs from the LH [37]. We found ipsilateral activation of the PVN by both drugs. However in the arcuate nucleus, ipsilateral activation of c-*fos* was only seen after baclofen infusion (Figure 7, Table 3). Baldo et al. [33] reported elevated c-*fos* expression in the arcuate after bilateral infusion of a substantially higher dose of muscimol than was used here. The ipsilateral activation of LH, PVN and arcuate by baclofen is consistent with an interpretation of the induced feeding behavior being similar to normal, homeostatic, feeding [17].There was an increase in c-*fos* activation ipsilaterally in CeA after baclofen infusion (Figure 8, Table 3). There is a projection of LH to CeA [38]–[40] and there is also evidence for a direct projection from the accumbens shell to CeA [30]. The possibility of greater activation of the CeA by baclofen is of interest because of the suggestion by Balleine and Killross [41] that the CeA encodes incentive salience. This proposal is supported by a recent study by Mahler and Berridge [42] which demonstrated that manipulation of CeA opioid transmission selectively enhanced the attractiveness of a prepotent stimulus in an autoshaping paradigm. Thus the interpretation offered here for the role of incentive salience in the behavior of baclofen treated animals may reflect the input from the CeA to the accumbens [43], which may itself be enhanced by the infusion of baclofen into the accumbens.

An alternative locus for differences between the effects of the two drugs is within the accumbens itself. The accumbens is a heterogeneous structure with a complex set of both afferents and efferents, which can be considered to form a number of different neuronal ensembles [44]. The ability of the accumbens to produce output signals which differentiate incentive salience and hedonic impact has recently been demonstrated by Smith et al [45]. They observed that the response of neurons in the ventral pallidum is modified by the infusion of either amphetamine or opiates into the accumbens. This finding opens up the possibility that baclofen may also modify the output of the accumbens, perhaps to the central nucleus of the amygdala, in such a way as to signal an increase in incentive salience for the conditioned stimulus.

Both GABA_A_ and GABA_B_ receptors are widely distributed in the striatum, and have been reported on both interneurons and projection neurons [46]–[48]. As elsewhere in the CNS, it seems probable that the metabotropic GABA_B_ receptors will be located both presynaptically, as autoreceptors and heteroreceptors, as well as on the postsynaptic cell, although in this case peri- and extra-synaptically [48]. GABA_B_ receptors can act in a neuromodulatory fashion by both presynaptic and postsynaptic mechanisms to regulate glutamate transmission [49]. In contrast, the GABA_A_ receptors are only located on postsynaptic cells [50], although some subtypes may be located extrasynaptically [51].

All the physiologically identifiable classes of striatal GABAergic interneurons have been shown to produce fast, potent, monosynaptic inhibition of MSNs [52] which would be consistent with action through GABA_A_ receptors especially at synapses located on the perikarya [53]. Infusion of muscimol could therefore completely inhibit MSN output as a result of its high affinity to GABA_A_ receptors [54]. In contrast, baclofen may be acting at the dendrites of the MSNs through both presynaptic inhibition of the glutamatergic input there [29] and postsynaptic effects including direct modulation of glutamatergic NMDA synapses [29], [49]. At moderate doses baclofen may decrease excitatory drive to both the interneurons and the MSNs rather than directly inhibiting the MSNs. Thus the two GABAergic agonists may produce different effects as a result of the more potent and over-riding effect of muscimol as compared to the more subtle impact of baclofen which could permit other inputs to the accumbens to continue to influence behavior. The interactions between glutamatergic inputs to the accumbens is itself complex [55]–[57] and the effects of baclofen may be due to neuromodulatory changes in this, possibly changing the balance between the interplay of cortex, amygdala and hippocampus.

In conclusion, we have shown that intra-accumbens stimulation of GABA_B_ receptors, but not GABA_A_ receptors, increases instrumental responding for food on a second order schedule of reinforcement. In addition we observed differential patterns of stereotypy and other abnormal behaviour at higher doses of either GABA_A_ or GABA_B_ agonists which, in the case of the GABA_B_ receptor agonist baclofen, were likely to be sufficient to limit further increases in instrumental responding. This represents some of the first evidence of a clear differentiation of the effects of intra-accumbens stimulation of these two subtypes of GABA receptor. It also provides the first evidence that stimulation of accumbens shell GABA_B_ receptors can enhance instrumental responding for food, most likely by increasing the incentive salience of discrete CS’s within the second order schedule that we used for these studies.

## Materials and Methods

### Ethics Statement

The experiments were carried out in full compliance with the Animals (Scientific Procedures) Act 1986. UK Home Office Project License 90/60755 was approved following review by the University of Sussex Local Ethical Review Committee.

### Animals

Three separate groups of naïve male Lister hooded rats (Harlan, United Kingdom, N = 12, 175–200 g initially) were used, one for each experiment. Holding and test rooms were maintained at controlled temperature (19–22°C) and humidity (45–55% saturation) with a 12 hr light/dark cycle. Animals had *ad libitum* access to water throughout the study but access to food was restricted (see below). They were housed in groups of three or four during the period of instrumental training and moved to individual housing at least one week prior to surgery.

### Instrumental Schedule

Food was restricted using a daily ration of 14–16 g per rat to maintain weight at 85%–90% of free feeding animals. The animals were then trained to respond on a second order schedule [18] using standard two lever operant cages (Paul Fray, Cambridge). Initial habituation to consumption of food pellets in the operant cage was provided on a 120 s random time schedule for 30 minutes (2 days). The animals were then progressively trained on an FR1 and then a FR5 schedule (4–8 days). Active and inactive levers were counterbalanced across individuals and the cue light was illuminated for 4 s both prior to and following pellet delivery. Further training (4–8 days) introduced the animals to the second order schedule in which the animals would press a lever 5 times (reinforced lever presses) to receive an 8 second light CS. Further lever presses while the light was illuminated had no programmed consequences (non-reinforced lever presses). During the first 5 minutes of the 30 minute test session the animals only received light CS’s (‘appetitive phase’). For the remainder of the session the animals received 5×45 mg Noyes AI grain pellets after 5 illuminations of the light CS and hence after 25 lever presses (‘consummatory phase’). Food was delivered 4 s after illumination of the light, which remained illuminated for a further 4 s. This schedule maintains high levels of responding throughout the test session, including the initial 5 minutes during which food reward is not provided [18]. This initial period of responding provides a measure of response rate that is not affected by either the physiological or behavioural consequences of food consumption. All lever presses were recorded and time-stamped.

### Surgery

Anaesthesia was induced with 4% isoflurane in 0.5 L/min N_2_O and 0.5 L/min O_2_, and then maintained by adjusting the isoflurane concentration to 1.5–2.5%. Thin wall 26 g, 16 mm stainless steel cannulae (Coopers Needleworks, UK) were implanted bilaterally aimed 2.2 mm dorsal to the target site in the accumbens shell using the coordinates anteroposterior (AP) +1.4 mm, mediolateral (ML) ±0.9 mm relative to bregma (β) and dorsoventral (DV) −5.8 mm relative to the flat skull surface [58]. The cannulae were secured with three small screws, Geristore dental resin and a cap of Simplex dental acrylic. Cannula patency was maintained by 33 g wire obdurators. The incision was treated with Cicatrin powder (GlaxoSmithKline, UK) and the animals were administered an antibiotic (oxytetracycline 10 mg/kg) and a non-steroidal analgesic (meloxicam 2 mg/kg) immediately, and then at 24 and 48 hours after surgery. These drugs were mixed into a small pot of palatable wet mash to which the animals had been accustomed prior to surgery.

### Drugs

The GABA_B_ agonist baclofen (Sigma, UK) and the GABA_A_ agonist muscimol (Sigma, UK) were dissolved in a vehicle of 0.9% sterile saline, initially at ten times the required highest dose. The pH of the baclofen solution was adjusted to 7.5 using sodium hydroxide. Doses (see below) were chosen on the basis of earlier reports [2], [5] and minimally effective doses were determined by several pilot experiments. Doses above those reported here were excluded because pilot experiments indicated significant side effects in this rat strain. Baclofen (880 pmol) induced a myorelaxant effect which had a relatively small impact on the animals capacity to eat from a food dish but did impair their ability to press an operant lever whereas muscimol (880 pmol) led to very significant, and contrasting, motor impairment including catalepsy and muscular rigidity.

### Infusions

On test days bilateral infusions of drug or vehicle were made simultaneously into the accumbens shell at a rate of 0.5 µl per side over 30 seconds (1 µl of drug solution infused in total). Injectors were left in for a further minute to allow diffusion of drug away from the tip. The infusions were given using 31 g stainless steel infusors which extended 2.2 mm beyond the tip of the guide cannulae to reach the target structure. These injectors were connected via number 10 PPE tubing to 10 µl Hamilton syringes. A microinfusion syringe pump model 802 (Model 802, Univentor, Malta) which held two syringes allowed bilateral infusions to be made simultaneously. Behavioural testing followed immediately after the infusions were completed.

### Experimental Procedures

We separately examined the effect of baclofen (experiments 1 and 2) or muscimol (experiment 3) on intake of freely available chow and on instrumental responding in the second order schedule. Experiment 1 consisted of an initial test of free intake using a single drug dose followed by tests of instrumental responding at varied doses of drug. Experiments 2 and 3 consisted of an initial test of free intake using a single drug dose, tests of instrumental responding at varied doses of drug and, finally, tests of free intake at the same set of doses. The latter procedure was chosen to reveal any marked change in responsiveness with repeated intra-cerebral drug infusions during the experimental sequence. Drug doses were allocated using a randomised ascending Latin square design.

In Experiment 1 prior to instrumental testing, free food intake was measured in two sessions using either vehicle or 440 pmol baclofen, allowing at least two days between test sessions. For instrumental testing the pre-satiated animals received infusions (in pairs) of saline vehicle (0), 110, 220 or 440 pmol of baclofen in a randomized order. Each instrumental test session was initiated immediately after drug infusion was completed for the pair. Between test sessions, animals were given at least one drug-free instrumental session to maintain baseline responding.

In Experiment 2 the dose range was extended and the initial intake tests used 660 pmol baclofen or saline. They were followed by three instrumental test sessions using doses of 0, 220 and 660 pmol baclofen and a further three intake tests using the same doses. The instrumental test sessions were videoed for later analysis and, following histological verification of the infusion site, the brains of five of these animals were processed to reveal c-*fos* immunoreactivity.

Experiment 3 used a similar design to Experiment 2 in order to explore the effects of equimolar doses of muscimol (0, 220, 440, 660 pmol) on instrumental responding and intake of freely available food. The initial intake tests were carried out using 660 pmol muscimol or saline and were followed by the instrumental test sessions and finally intake tests at the same doses. The instrumental test sessions were videoed for later analysis. For both experiments 2 and 3, the behavioural categories were chosen after preliminary screening of the recordings and definitions are provided in the Legend to Table 2. Aversive responses, such as defensive treading, were absent. As before, the brains of five of these animals were processed to reveal c-*fos* immunoreactivity.

### Perfusion and Dissection

At the end of each experiment subjects were deeply anaesthetised and transcardially perfused with phosphate buffered saline followed by 10% formol saline (experiment 1) or 4% paraformaldehyde (experiments 2 & 3). Two hours prior to perfusion, animals were unilaterally infused with 0.5 µl of either 220 pmol/µl baclofen (Experiment 2) or 220 pmol/µl muscimol (Experiment 3) and 0.5 µl vehicle on the contralateral side. After perfusion, the heads were left in fixative for at least 24 hours before dissection was completed and the brains returned to fixative for a further 24 hours. The brains were then cryoprotected in phosphate buffered 30% sucrose solution by immersing them for a minimum of 48 hours prior to freezing and storage at −80°C.

### Histology and Placement Verification

Brains were sectioned coronally at 60 µm on a freezing microtome. Every 5^th^ section from the nucleus accumbens was mounted on gelatinized slides stained with thionin. By comparing key landmarks with the appropriate figures in Paxinos and Watson [58], the location of infusion sites was determined. Gliosis could be seen around both the cannula and around the infusion site itself. By using the length of protusion of the infusor (2.2 mm) the infusion site could be placed within the lower area of gliosis. A correct placement was defined as being within the shell region mediolaterally and between anterior-posterior co-ordinates of 1.4 mm ±0.5 mm. Animals with gliosis that appeared to have spread too extensively dorsoventrally (into other brain structures) were also excluded from the final group for analysis.

### Tissue Preparation for cFos Immunocytochemistry

After evaluation of cannula placements, additional sections from 5 selected animals were processed to reveal c-*fos* immunoreactivity. Five brains were selected for each drug treatment and were chosen on the basis of how close the infusion sites were to the target co-ordinates and also on the basis that a minimal amount of visible gliosis was present.

These 10 brains were sectioned coronally at 40microns on a freezing microtome and the tissue floated in 0.9% physiological saline. Every 6th section from relevant areas of the brain (see Table 3) were mounted and stained with thionin. These were later used to identify landmarks for structural identification of areas of Fos positive nuclei present on the remaining sections. In total sections were cut from the point at which the anterior commissure crosses the midline of the brains at approximately bregma +0.25 mm to approximately bregma −5.8 mm.

### Method for Immunohistochemical Staining

The procedures used here are based on a protocol described by Elmquist et al. [59] and adapted for use with free floating rat brain sections on the basis of a protocol described by Kalinichev et al. [60]. Staining always included one brain from each of experiments 2 and 3. Three sections from each brain were used as negative controls for non-specific binding (omission of primary antibody. Unless otherwise stated all washing and incubation stages described below took place at room temperature on an orbital shaker. The immuno-buffer was Tris buffered PBS/0.3% Triton X-100 solution, pH 7.4.

The staining protocol was as follows:

1. Wash in immuno-buffer for 15 minutes.
2. Incubate for 30 minutes in 1% hydrogen peroxide.
3. Two 10 minute washes in immuno-buffer.
4. Block non-specific antibody binding by incubating the sections for 1 hour in 2% goat serum (Sigma, UK) diluted in immuno-buffer.
5. Incubate for approximately 48 hours at 4°C to 2 mls per well of the primary (1°) antibody (a 1∶20,000 dilution of Anti-c-Fos (Ab-5) Rabbit pAb in immuno-buffer, Calbiochem supplied by Merck Chemicals Ltd., UK).
6. Bring sections up to room temperature.
7. Three 10 minute washes in immuno-buffer.
8. Incubate for 2 hours in 0.8 ml per well of the secondary (2°) antibody (a 1∶20,000 dilution of biotinylated goat anti-rabbit IgG (H+L) in immuno buffer, Vector Laboratories, USA).
9. Three 10 minute washes in immuno-buffer.
10. Incubate with 0.6 mls of ABC reagent for 2 hours.
11. Three 10 minute washes in a Tris-0.84 M HCl buffer (Tris-HCl), pH 7.4.
12. Incubate with 0.6 ml of the DAB solution for 10 minutes.
13. Wash for 10 minutes with Tris-HCl buffer.
14. Wash in 0.9% physiological saline.

After this, sections were mounted from physiological saline onto gelatinised slides. Slides were air dried and the sections fixed in formaldehyde vapour for 1 hour. Sections were cleared in Histoclear (R.A. Lamb, UK) then cover slipped with Histomount (R.A. Lamb, UK). Once the mounting agent had fully set, slides were cleaned and polished with IMS.

### ABC and DAB Solutions

Horseradish peroxidase avidin-biotin complex (ABC) reagent from an Elite standard peroxidase ABC kit (Vector Laboratories, USA) was prepared according to manufacturer’s instructions. Diaminobenzidine (DAB; 0.035%) was made up using a DAB Kit for peroxidase (Vector Laboratories, USA) following the manufacturer’s instructions. The nickelammonium sulphate solution was excluded thus giving a brown coloured product which gave a good contrast for subsequent image analysis.

### Image Analysis and Quantification

Tissue sections were viewed with a Zeiss Akioskop 2 plus microscope and images of sections were captured using an AxioCam HRc digital camera (Carl Zeiss, UK) using AxioVision 3.1 software (Imaging Associates, Bicester, UK). Coordinates for areas of interest were taken from the Paxinos and Watson [58] as shown in Table 3, following those given by Stratford [18] where specified. For the ventral pallidum, basolateral nucleus of the amygdala and ventral tegmental area, the precise co-ordinates were chosen on the basis of the highest level of staining within the sections through the relevant structure for each animal. Sections for each brain area were matched across the animals and a thionin stained section selected as the basis for a template which could be superimposed onto the Fos stained sections.

Counting was automated using ImageJ 1.4 for Mac (NIH). A subgroup of sections from each region of the brain was also counted by eye to check the accuracy of the automated counting. This confirmed that there was never any more than a 1% difference between automated counts and counts made by eye.

### Statistics

Food intake, instrumental responses and cell counts were expressed as means (±SEM) and analysed using ANOVA with dose as a repeated measure factor. Each of the eight mutually exclusive behaviour patterns recorded during experiments 2 and 3 were treated separately. Paired comparisons between vehicle and drug conditions were made using Dunnett’s test and other paired comparisons used Bonferroni’s method. Statistical analysis was carried out using the Genstat 13 statistical package.
